# Parental Educational Attainment, the Superior Temporal Cortical Surface Area, and Reading Ability among American Children: A Test of Marginalization-Related Diminished Returns

**DOI:** 10.3390/children8050412

**Published:** 2021-05-18

**Authors:** Shervin Assari, Shanika Boyce, Mohsen Bazargan, Alvin Thomas, Ryon J. Cobb, Darrell Hudson, Tommy J. Curry, Harvey L. Nicholson, Adolfo G. Cuevas, Ritesh Mistry, Tabbye M. Chavous, Cleopatra H. Caldwell, Marc A. Zimmerman

**Affiliations:** 1 Minorities’ Diminished Returns (MDRs) Center, Charles R. Drew University of Medicine and Science, Los Angeles, CA 90059, USA; ShanikaBoyce@cdrewu.edu (S.B.); Mohsenbazargan@cdrewu.edu (M.B.); 2 Department of Urban Public Health, Charles R. Drew University of Medicine and Science, Los Angeles, CA 90059, USA; 3 Department of Family Medicine, Charles R. Drew University of Medicine and Science, Los Angeles, CA 90059, USA; 4 Department of Pediatrics, Charles R. Drew University of Medicine and Science, Los Angeles, CA 90059, USA; 5 Department of Family Medicine, University of California-Los Angeles (UCLA), Los Angeles, CA 90095, USA; 6 Human Development and Family Studies Department, School of Human Ecology, University of Wisconsin-Madison, Madison, WI 53706, USA; athomas42@wisc.edu; 7 Department of Sociology, University of Georgia, Athens, GA 30602, USA; ryon.cobb@gmail.com; 8 Brown School, Washington University, St. Louis, MO 63130, USA; d.hudson@wustl.edu; 9 Department of Philosophy, School of Philosophy, Psychology and Language Sciences, University of Edinburgh, Edinburgh EH8 9JS, UK; t.j.curry@ed.ac.uk; 10 Department of Sociology and Criminology & Law, University of Florida, Gainesville, FL 32611-7330, USA; hnicholson@ufl.edu; 11 Psychosocial Determinants of Health (PSDH) Lab, Tufts University, Boston, MA 02155, USA; adolfo.cuevas@tufts.edu; 12 Department of Community Health, Tufts University, Boston, MA 02155, USA; 13 Department of Health Behavior and Health Education, University of Michigan School of Public Health, Ann Arbor, MI 48109-2029, USA; riteshm@umich.edu (R.M.); cleoc@umich.edu (C.H.C.); marcz@umich.edu (M.A.Z.); 14 School of Education, University of Michigan, Ann Arbor, MI 48109-2029, USA; tchavous@umich.edu; 15 National Center for Institutional Diversity, University of Michigan, Ann Arbor, MI 48109-2029, USA; 16 Department of Psychology, University of Michigan, Ann Arbor, MI 48109-2029, USA; 17 Center for Research on Ethnicity, Culture, and Health (CRECH), University of Michigan School of Public Health, Ann Arbor, MI 48109-2029, USA; 18 Prevention Research Center, University of Michigan School of Public Health, Ann Arbor, MI 48109-2029, USA

**Keywords:** socioeconomic factors, population groups, brain development, reading, school performance, cortical surface, child, adolescents, magnetic resonance imaging

## Abstract

Background: Recent studies have shown that parental educational attainment is associated with a larger superior temporal cortical surface area associated with higher reading ability in children. Simultaneously, the marginalization-related diminished returns (MDRs) framework suggests that, due to structural racism and social stratification, returns of parental education are smaller for black and other racial/ethnic minority children compared to their white counterparts. Purpose: This study used a large national sample of 9–10-year-old American children to investigate associations between parental educational attainment, the right and left superior temporal cortical surface area, and reading ability across diverse racial/ethnic groups. Methods: This was a cross-sectional analysis that included 10,817 9–10-year-old children from the Adolescent Brain Cognitive Development (ABCD) study. Parental educational attainment was treated as a five-level categorical variable. Children’s right and left superior temporal cortical surface area and reading ability were continuous variables. Race/ethnicity was the moderator. To adjust for the nested nature of the ABCD data, mixed-effects regression models were used to test the associations between parental education, superior temporal cortical surface area, and reading ability overall and by race/ethnicity. Results: Overall, high parental educational attainment was associated with greater superior temporal cortical surface area and reading ability in children. In the pooled sample, we found statistically significant interactions between race/ethnicity and parental educational attainment on children’s right and left superior temporal cortical surface area, suggesting that high parental educational attainment has a smaller boosting effect on children’s superior temporal cortical surface area for black than white children. We also found a significant interaction between race and the left superior temporal surface area on reading ability, indicating weaker associations for Alaskan Natives, Native Hawaiians, and Pacific Islanders (AIAN/NHPI) than white children. We also found interactions between race and parental educational attainment on reading ability, indicating more potent effects for black children than white children. Conclusion: While parental educational attainment may improve children’s superior temporal cortical surface area, promoting reading ability, this effect may be unequal across racial/ethnic groups. To minimize the racial/ethnic gap in children’s brain development and school achievement, we need to address societal barriers that diminish parental educational attainment’s marginal returns for middle-class minority families. Social and public policies need to go beyond equal access and address structural and societal barriers that hinder middle-class families of color and their children. Future research should test how racism, social stratification, segregation, and discrimination, which shape the daily lives of non-white individuals, take a toll on children’s brains and academic development.

## 1. Background

Researchers have documented large, sustained racial/ethnic gaps in school performance and academic achievement in the United States [1,2,3,4]. Compared to white and Asian children, black, American Indian, Alaskan Native, Native Hawaiian, and Pacific Islander (AIAN/NHPI) children fare poorer in various school performance domains [5,6]. Jim Crow policies created high levels of racial segregation throughout the US that persist to this day. Racial and ethnic inequalities in school performance have emerged and deteriorated due to the neighborhood and school segregation [7,8,9]. School quality is substantially worse for children from racial and ethnic minority backgrounds and low socioeconomic status (SES) [10]. Furthermore, children from historically marginalized communities are exposed to greater levels of financial difficulties and psychosocial stressors [11] that results in lower memory, cognitive function, behavior, and school performance [12]. Additionally, greater exposure to indoor and outdoor toxins, such as air pollutants and lead [13], deteriorate the brain development of racial and ethnic minority brain development. Due to these social and physical environmental factors, researchers have observed lower academic achievement for racial and ethnic minorities [14]. The black–white achievement gap, for example, is a very well described phenomenon in the fields of education and psychology and has been sustained for centuries due to historical injustice and lack of political will to undo them [15,16].

Although academic disparities exist for math and science performance [17,18,19], racial and ethnic disparities in reading ability are of particular interest [20]. Here, we list five reasons: First, these differences are substantial in size. According to the National Assessment for Education Progress (NAEP) report, black students’ reading abilities (grades) were three-quarters of a standard deviation below white students’ scores, which is equal to a difference of four years of learning [21]. According to the Annie E. Casey Foundation, white students are three times more likely to be proficient at reading among fourth-graders than their black peers [22,23]. Second, the black–white reading gap has been stable since the 1970s [24,25]. Third, the reading gap has long-term consequences. Reading ability in elementary school is a strong predictor of reading ability 10 years later [26]. Reading is core to learning, and low reading ability has spill-over effects on almost all school achievement domains [27]. This is consistent with the observation that reading ability mediates the effect of parental education on neurocognitive abilities, such as executive functioning [28]. Fourth, the brain regions behind reading ability are well-established (e.g., the superior temporal cortex), making reading ability an ideal outcome variable in the neuroscience of children’s brain development [29,30,31]. Finally, some evidence suggests that the racial gap in children’s reading ability may explain some of the diminishing returns of SES for black rather than white adults later in life [32,33]. Reading is a good indicator of academic achievement and has strong connections to race/ethnicity, SES, brain development, and future health and economic outcomes. These reasons collectively make studying the associations between race/ethnicity, parental education, the superior temporal cortex, and reading ability a unique opportunity that can help us understand how race/ethnicity, SES (parental education), and brain development interact and shape racial and ethnic gaps in school performance, particularly for middle-class families.

The superior temporal cortical surface area is commonly studied in brain imaging of reading ability [34,35,36,37]. Research has already established the effects of SES and race/ethnicity effects on this brain region as an underlying mechanism that helps us understand how race and SES contribute to social inequalities in reading abilities [38,39]. The superior temporal cortex’s role in the comprehension of sentence meaning and structure [39], as well as naming words [35], is well established. As such, the superior temporal cortical surface area can be seen as a candidate brain region that can explain social inequalities in children’s reading ability [35,40]. A recent study by Merz, Noble, et al. [34] used structural magnetic resonance imaging (MRI) (sMRI) data, as well as home-observation of family interactions, and explored associations between family SES, home linguistic input, left perisylvian cortical surface area (e.g., the superior temporal cortex), and reading skills among five to nine year-old children. Authors analyzed data of naturalistic home audio recordings in addition to T1-weighted MRI scans of the greater left perisylvian (e.g., the superior temporal cortex) cortical surface area. The study showed that family SES was associated with parent–child conversational turns, as well as parent words, which in turn were linked to a larger cortical surface area in areas such as the superior temporal cortex. Interestingly, language input partially mediated the effect of parental education on the left surface area in brain regions such as the superior temporal cortex (i.e., left perisylvian cortical surface area). Similarly, the left perisylvian surface area mediated the association between parental education and children’s reading skills. Merz et al. [34] concluded that morphometric brain features, such as the superior temporal cortex area, may be one of the mechanisms that explain why children with highly educated parents have better reading abilities. The researchers suggest that this is partly because more educated parents talk more frequently with their children, who get more opportunities to discuss ideas than children with lower educated parents. These bidirectional conversations enhance children’s language experiences and associated brain area, which in turn predicts children’s reading skills [34].

While parental education, the superior temporal cortex, and reading ability are linked, recent research by Assari and colleagues have reported that the magnitude of the associations between parental education (and other SES indicators), brain structures (e.g., cortical surface area), and behavioral outcomes are unequal across race/ethnic groups [41]. Marginalization-related diminished returns (MDRs) refer to systematically weaker effects of SES resources, particularly parental education, on a wide range of health and developmental outcomes of children from marginalized families compared to privileged families [42]. As a result of such MDRs [43,44,45,46], black children from high SES families remain at risk of smoking [47], aggression [48], depression [49], suicide [50], internalization [51,52], externalization [51,52], and poor school functioning [48] compared to white children with identical SES. High SES black children also show worse than expected grades in reading, math, and science than their white counterparts [53,54]. This pattern is not specific to black children as it is also shown for children from AIAN/NHPI, Latino, Asian American, and even marginalized white families [53,54,55,56,57].

In the Monitoring the Future (MTF) study, high parental education was associated with a larger enhancement of school performance for white children than black and Latino children [53]. While in the pooled sample and in white participants, we found an apparent step-wise increase in youth school performance with higher parental education, this step-wise improvement was absent for black and Hispanic youth [53]. We also observed similar findings in college students from the Healthy Mind Study (HMS, 2016), which showed that parental education had a weaker effect on the GPA of black than white college students [55]. In another study, using data from the Population Assessment of Tobacco and Health (PATH 2013–2014) data, we found that, although high parental educational attainment was predictive of a higher GPA, race and ethnicity showed significant interactions with parental educational attainment on students’ GPA. As such, Hispanic and black children with highly educated parents still had lower GPAs, while white youth with the same parental education showed a higher gain in terms of GPA due to their parental education [54]. All these studies show that, across samples and age groups, high parental education promotes school performance of white more than black children and youth (i.e., MDRs).

In line with the MDRs described above, our analysis of the Adolescent Brain Cognitive Development (ABCD) data shows that the effects of SES indicators, particularly parental education, on brain function and structure are weaker for black than white children. For example, the effects of parental education on attention, working memory, and inhibitory control are all weaker for black than white children [58,59,60]. Analysis of the sMRI and fMRI data from the same sample also indicates that SES effects on hippocampus and amygdala volume [61] and the cortical surface area [41] were weaker for black than white children. Similarly, some of the effects of age on brain function [62,63] were weaker in black than white children. These observations are consistent with weaker SES effects on behavioral (externalizing) and mental health (depression, suicide, and stress) outcomes for black than white children [50,51,64]. We are not aware of any studies conducted explicitly on differential associations between SES, the superior temporal cortical surface area, and reading ability by race/ethnicity. Thus, there is a need to test the relevance of MDRs for the associations between parental education, the superior temporal cortical surface area, and reading scores among American children.

Since any research in the field of race, SES, brain, and cognitive function has the potential to be misinterpreted, we emphasize that MDR theory does not attribute these racial and ethnic disparities in SES effects on brain development and cognitive outcomes to biology. Instead, MDRs attribute them to social forces, historical injustice, and inaction to undo these disparities. Thus, MDR research takes a social rather than biological perspective to explain the observed disparities. These MDRs are shaped by contextual factors that undermine marginalized people’s life chances, broadly defined to include immigrants, Latino, Asian, AIAN/NHPI, and black individuals. The racial and SES gap in school performance is due to contextual and social factors, not immutable biological differences across race/ethnic groups. As these patterns are shown in various racial and ethnic minorities, but also immigrants [64,65], LGBT [66,67], and even marginalized white people [57], suggesting that any type of social marginalization reduces the gains of SES, black communities should not be blamed for undesired outcomes. These gaps are believed to be due to social, contextual, and environmental (modifiable) factors, such as low school quality in urban areas [68], residential segregation [69], and labor market discrimination [45], which all reduce the return of educational attainment for black communities. Beyond the work by our team [43,44], other scholars, such as Hudson [70], Thorpe [71], Williams [72], Ferraro [73], and others [74], have also described the same phenomenon, showing that SES effects tend to be weaker for non-whites than whites. However, most of that literature has not used the term MDRs.

## 2. Aims

Built on the MDRs phenomenon overall [44,45,75,76], MDRs of parental education on cognitive ability [60,62,63,77], school performance [53,54,55,57], and brain structures [41] in particular, we used data from Adolescent Brain Cognitive Development (ABCD) study [75,76,77,78,79,80,81,82] to compare the associations between parental educational attainment, the superior temporal cortical surface area, and readability across diverse racial/ethnic groups in a large, diverse national sample of American children. In line with a recent work by Merz [34], we hypothesized positive associations between parental educational attainment, the superior temporal cortical surface area, and children’s reading ability. However, guided by an MDR perspective [41], we hypothesized weaker associations between these constructs in non-white than white children. In other terms, we expected parental education to be associated with a smaller increase in the superior temporal cortical surface area and the reading ability for non-white than white children. Similarly, based on the MDRs, we expected an increase in the superior temporal cortical surface area associated with a smaller increase in reading ability for non-white than white children. For two reasons, we expect more pronounced differences between black and white children than other racial and ethnic groups: First, because research has demonstrated stronger MDRs for black individuals than other non-white groups [44,45,76], and second, because the history of oppression and suppression in black communities is not comparable with the experiences of other groups [83,84].

## 3. Methods

Our cross-sectional study used data from wave 1 of the ABCD study [75,76,77,78,79,80,81,82]. ABCD is a state-of-the-art study of brain development of 9–10-year-old American children [75,76,77,78,79,80,81,82]. The ABCD study includes a diverse sample regarding race/ethnicity, SES, and family structure. Although the sample is not nationally representative, the ABCD study has used sampling strategies to increase the results’ generalizability to the US sample [80]. Although ABCD has a national sample that is recruited from 21 sites across 15 states, the sample is not nationally representative.

The ABCD sample is mainly recruited from schools [80]. To increase the number of non-white participants, particularly black children, recruitment also occurred outside school contexts in collaboration with local community organizations [80]. Comparing racial/ethnic groups in the ABCD sample requires attention to these different sampling strategies. Nevertheless, over 100 papers support MDRs across national and local studies, with various sampling designs. Thus, issues with sampling design should not be consequential as MDRs are robust regardless of the sampling design [85].

Participants were enrolled between 2016 and 2018 by 21 ABCD sites that were located in the following 15 states: Maryland, California, Colorado, Connecticut, Florida, Michigan, Minnesota, Missouri, New York, Oklahoma, Oregon, Pittsburgh, South Carolina, Utah, Vermont, Virginia, and Wisconsin. The number of participants across each site is shown in Appendix C. The number of participants enrolled over time is shown in Appendix D.

### 3.1. Measures

This study used data on demographics, race/ethnicity, SES, sMRI, and reading tasks. Our demographic variables included age, sex, family structure, and parental education, and our sMRI data was limited to the superior temporal cortical surface area. Reading was measured using NIH Toolbox [86,87].

*Race.* This study conceptualized race as a social construction, which means we see race as not based on some innate and immutable scientific fact, but rather, the social meanings ascribed to racial categories. In this view, race is a proxy of racism, adversity, and living conditions, rather than genetics or biological differences.

*Parental education.* Parents reported the highest education level in their household, and this included the following categories: Less than high school, high school completed, some college, college graduated, and graduate studies. This variable captured both maternal and paternal education. This variable was a nominal variable, with less than high school as the reference group.

### 3.2. Scanning Protocol

Structural MRI was performed at 21 sites in the United States using a standardized protocol for imaging acquisition, processing, reconstruction, and quality control [77]. All structural MRI findings were screened for incidental findings by a neuroradiologist [77]. A full description of image acquisition and processing is available elsewhere [77]. Although the ABCD study has functional and diffusion MRI data, this study only used structural neuroimaging data (sMRI). In the ABCD study data, whole-brain coverage was achieved with the following indicators and features: isotropic voxel resolution of 1 × 1 × 1 × mm, 256 × 256 matrices, flip angle of 8°, an inversion delay of 1060 milliseconds, 176 to 225 sections, the field of view of 256 × 240 to 256, the field of view phase of 93.75% to 100%, repetition time of 2400 to 2500 milliseconds, echo time of 2 to 2.9 milliseconds, and parallel imaging of 1.5 × 2.2. The total image acquisition time varied between 5 min and 38 s to 7 min and 12 s [77].

### 3.3. Image Reconstruction

The ABCD study team generated structural MRI data from T1-weighted and T2-weighted images. These data were corrected for gradient non-linearity distortions to ensure reliability across multiple imaging sites [88]. Intensity nonuniformity correction was based on tissue segmentation and sparse spatial smoothing. The ABCD study team then resampled images with 1-mm isotropic voxels into rigid alignment within the brain atlas. The cerebral cortex was then reconstructed, and volumetric segmentation was performed using FreeSurfer software, version 5.3.0 (Harvard University, Cambridge, MA, USA) [89,90]. Skull and nonbrain material were stripped from images. White matter and gray matter segmentation were performed by the ABCD study team using mesh creation. Topologic defects were corrected using the procedures described by Fischl et al. [91] and Ségonne et al. [92]. Images underwent surface optimization [93] and nonlinear registration to a spherical surface-based atlas [94]. The ABCD team then parcellated and labeled the cortical regions using a surface-based atlas classification that provides brain region of interest (ROIs)-level results [95]. The ABCD study team conducted all the procedures described above, and the ROI data are freely available within the data releases. We also used pre-processed MRI data, which is available in the released ABCD data files. The only sMRI data used for this analysis was the superior temporal cortical surface area. This was a continuous variable in mm^3^, with a higher score indicating a larger surface area of our ROI.

### 3.4. Imaging Quality Control

A detailed harmonization process was used to maximize similarity in the process across scanning sites. As such, ABCD study has heavily relied on comprehensive quality control and harmonization protocol across imaging sites to ensure the integrity and comparatively of the brain imaging data [77]. Images were manually reviewed for data quality. Images with the most severe artifact, irregularities, and/or poor image quality were rejected and excluded from processing and analysis. Cortical surface reconstruction images were reviewed for motion, intensity inhomogeneity, white matter underestimation, pial overestimation, magnetic susceptibility artifacts, and susceptibility artifacts [77].

### 3.5. Reading Ability

The ABCD study uses a wide range of cognitive measures [86,87]. Reading ability was measured using the NIH Toolbox Oral Reading Recognition Test Age 3+ v2.0 Uncorrected Standard Score. The NIH reading measure is one of NIH toolbox’s cognitive performance batteries [96]. These measures are highly reliable and valid [97] and widely used to assess reading [98] and other cognitive function aspects of children and adults [99]. The reading measure provides a continuous variable, with a higher score reflecting higher reading ability and comprehension of words, text, and sentence structures [100].

## 4. Data Analysis

We conducted the data analysis in the Data Exploration and Analysis Portal (DEAP). DEAP is an online platform based on the R-package, specifically designed for the analysis of ABCD data. Given the nested nature of the ABCD data, DEAP applied a mixed-effects regression model for data analysis. We calculated and reported regression coefficients for the following models: *Model 1*, the Main Effect model, did not include any interaction terms. *Model 2*, the interaction model, did include interaction terms between race/ethnicity and our independent variables. Thus, *Model 2* was *Model 1* plus interaction terms. These models were run for (1) the effects of parental education on the superior temporal cortical surface area, (2) the effects of the superior temporal cortical surface area on reading ability, and (3) the effects of parental education on reading ability. We did not adjust for multiple comparisons because we did not run exploratory models, did not have specific hypotheses for the associations tested, and our main goal was to test statistical interaction, which requires more power than regular analysis. Appendix A shows our modeling strategy for the three aims listed above. Appendix B show the results of our tests of assumptions, such as normality of the error terms for our outcomes and our models. We limited our sample to those with valid data on our variables of interest for our primary statistical analysis, but we did not limit our sample to any specific race/ethnicity. We ran models in the pooled sample and sub-sample that met restrictive quality control of the imaging for our sensitivity analysis. As results did not change, we reported the results with larger sample size. Without showing the numbers, our sensitivity analyses and robustness check results are mentioned at the end of the results section.

## 5. Results

### 5.1. Descriptive Data

Our descriptive statistics are shown in Table 1. This table shows the distribution of demographics, SES (parental education), the superior temporal cortical surface area, and reading ability for the overall sample and for racial/ethnic groups. This sample was mainly white (*n* = 7091; unweighted 65.6%; weighted 68.3%), followed by a mixed (*n* = 1298 unweighted 12.0% weighted 7.5%) racial group. The remaining sample was either black (*n* = 1646; unweighted 15.2%; weighted 13.9%), other race (*n* = 468; unweighted 4.3%; weighted 5.3%), Asian (*n* = 246; unweighted 2.3%; weighted 3.7%), or AIAN/NHPI (*n* = 68 unweighted 0.6%; weighted 1.2%).

Table 1 also reports the results of ANOVA and Chi-square to compare the study variables across race/ethnicity groups. As this table shows, Asian and white children had higher parental education than black, other-race, and mixed-race children. Additionally, Asian and white children had a higher reading ability, and black, other-race, and mixed-race children had a lower reading ability. Similarly, Asian and white children were more likely to have married families, and black, AIAN/NHPI, other-race, and mixed-race children were less likely to have married families. Black, other-race, and mixed-race children had smaller right and left superior temporal cortical surface areas and white children had a larger right and left superior temporal cortical surface areas.

### 5.2. Model Fit

As mentioned before, *Model 1,* the Main Effect model, did not include any interaction terms. *Model 2,* the interaction model, did include interaction terms between race/ethnicity or our independent variables. Thus, *Model 2* was *Model 1* plus interaction terms. Our models fit statistics and showed a better fit in the presence of statistical interaction terms, regardless of the association that was being tested. This suggests that, regardless of the outcome, the inclusion of interactions between race/ethnicity and our predictors always explained more of the outcome’s variance than that without the inclusion of interaction terms (Table 2).

### 5.3. Main Effects (Overall Effects)

Table 3, Table 4, Table 5 and Table 6 show our models without interaction terms (*Model 1*, right panel) and models with interaction terms (*Model 2*, left panel), both in the overall sample. As shown in the rights panels of these tables, there were positive associations between parental educational attainment, children’s right superior temporal cortical surface area, children’s left superior temporal cortical surface area, and children’s reading ability. To be more specific, the results confirm these hypotheses: (1) higher parental educational attainment is associated with larger right and left superior temporal cortical surface areas in children, (2) larger right and left superior temporal cortical surface areas are associated with higher reading ability in children, and (3) higher parental educational attainment is positively associated with higher reading ability in children. All these models adjusted for the nested nature of the data and controlled for covariates.

### 5.4. Interaction Effects (Heterogeneity of the Effects by Race/Ethnicity)

Table 3, Table 4, Table 5 and Table 6 also show the results of models with the interaction terms in the right panel. As shown in these tables, there were significant interactions between race/ethnicity and our predictors on our overall sample outcomes. In most cases, the statistical significance of the interaction terms was indicative of differences in the slopes for black, AIAN/NHPI, other-race, or mixed-race children relative to white children. The positive associations were weaker for black, AIAN/NHPI, other-race, or mixed-race children relative to their white counterparts. The exception was the association between parental education of reading ability, which was stronger for black than white children (reverse interaction was found).

However, Table 7 shows an interaction that is opposite to the interactions described above. We found a positive association between high parental education and reading ability, which was stronger in black than white children.

**Table 3:** Summary of mixed-effects regression models on the association between parental education and the right superior temporal cortical surface area.

	b	SE	*p*	sig	b	SE	*p*	sig
	Model 1				Model 2			
**Outcome**: Right Superior Temporal Cortical Surface Area								
Parental Education (HS Diploma/GED)	35.86	23.16	0.122		88.31	35.33	0.012	*
Parental Education (Some College)	71.15	20.93	0.001	***	122.72	30.55	<0.001	***
Parental Education (Bachelor)	118.21	21.78	<0.001	***	183.71	30.63	<0.001	***
Parental Education (Post Graduate Degree)	170.79	21.67	<0.001	***	237.53	30.37	<0.001	***
Age (Month)	−1.02	0.50	0.043	*	−1.01	0.51	0.045	*
Sex (Male)	264.53	7.83	<0.001	***	265.47	7.84	<0.001	***
Married Family	12.23	10.13	0.227		14.10	10.15	0.165	
Race/Ethnicity (Black)	−141.71	13.80	<0.001	***	−36.91	46.41	0.426	
Race/Ethnicity (Asian)	−123.16	25.70	<0.001	***	−96.23	142.54	0.500	
Race/Ethnicity (AIAN/NHPI)	13.46	46.87	0.774		−221.73	162.06	0.171	
Race/Ethnicity (Other)	−100.33	20.61	<0.001	***	5.91	50.17	0.906	
Race/Ethnicity (Mixed)	−44.54	14.78	0.003	**	147.04	74.65	0.049	*
Parental Education (HS Diploma/GED) × Race/Ethnicity (Black)					−83.31	54.48	0.126	
Parental Education (Some College) × Race/Ethnicity (Black)					−88.15	50.03	0.078	#
Parental Education (Bachelor) × Race/Ethnicity (Black)					−134.58	55.56	0.015	*
Parental Education (Post Graduate Degree) × Race/Ethnicity (Black)					−161.81	56.43	0.004	**
Parental Education (HS Diploma/GED) × Race/Ethnicity (Asian)					28.99	230.69	0.900	
Parental Education (Some College) × Race/Ethnicity (Asian)					46.65	167.17	0.780	
Parental Education (Bachelor) × Race/Ethnicity (Asian)					−44.80	150.31	0.766	
Parental Education (Post Graduate Degree) × Race/Ethnicity (Asian)					−33.20	146.11	0.820	
Parental Education (HS Diploma/GED) × Race/Ethnicity (AIAN/NHPI)					335.45	185.50	0.071	#
Parental Education (Some College) × Race/Ethnicity (AIAN/NHPI)					221.71	178.12	0.213	
Parental Education (Bachelor) × Race/Ethnicity (AIAN/NHPI)					189.23	191.30	0.323	
Parental Education (Post Graduate Degree) × Race/Ethnicity (AIAN/NHPI)					669.07	281.32	0.017	*
Parental Education (HS Diploma/GED) × Race/Ethnicity (Other)					−134.97	66.83	0.043	*
Parental Education (Some College) × Race/Ethnicity (Other)					−69.39	60.12	0.248	
Parental Education (Bachelor) × Race/Ethnicity (Other)					−166.31	72.22	0.021	*
Parental Education (Post Graduate Degree) × Race/Ethnicity (Other)					−170.03	79.18	0.032	*
Parental Education (HS Diploma/GED) × Race/Ethnicity (Mixed)					−188.38	90.57	0.038	*
Parental Education (Some College) × Race/Ethnicity (Mixed)					−175.63	78.41	0.025	*
Parental Education (Bachelor) × Race/Ethnicity (Mixed)					−198.47	80.36	0.014	*
Parental Education (Post Graduate Degree) × Race/Ethnicity (Mixed)					−222.69	79.69	0.005	**

# *p* < 0.1, * *p* < 0.05, ** *p* < 0.01, *** *p* < 0.001.

**Table 4:** Summary of mixed-effects regression models on the association between parental education and the left superior temporal cortical surface area.

	b	SE	*p*	sig	b	SE	*p*	sig
	Model 1				Model 2			
**Outcome**: Left Superior Temporal Cortical Surface Area								
Parental Education (HS Diploma/GED)	46.29	25.59	0.071	#	76.17	39.05	0.051	#
Parental Education (Some College)	81.02	23.13	<0.001	***	106.93	33.77	0.002	**
Parental Education (Bachelor)	136.35	24.07	<0.001	***	183.84	33.87	<0.001	***
Parental Education (Post Graduate Degree)	185.33	23.95	<0.001	***	232.14	33.57	<0.001	***
Age (Month)	0.35	0.55	0.525		0.37	0.55	0.506	
Sex (Male)	368.59	8.62	<0.001	***	369.85	8.62	<0.001	***
Married Family	8.97	11.19	0.423		11.60	11.21	0.301	
Race/Ethnicity (Black)	−143.15	15.23	<0.001	***	−50.34	51.25	0.326	
Race/Ethnicity (Asian)	−146.29	28.43	<0.001	***	−352.68	156.40	0.024	*
Race/Ethnicity (AIAN/NHPI)	−41.51	51.88	0.424		−343.02	179.68	0.056	#
Race/Ethnicity (Other)	−106.76	22.77	<0.001	***	−35.54	55.38	0.521	
Race/Ethnicity (Mixed)	−49.96	16.27	0.002	**	68.54	82.16	0.404	
Parental Education (HS Diploma/GED) × Race/Ethnicity (Black)					−66.00	60.14	0.272	
Parental Education (Some College) × Race/Ethnicity (Black)					−60.76	55.24	0.271	
Parental Education (Bachelor) × Race/Ethnicity (Black)					−160.15	61.31	0.009	**
Parental Education (Post Graduate Degree) × Race/Ethnicity (Black)					−150.43	62.24	0.016	*
Parental Education (HS Diploma/GED) × Race/Ethnicity (Asian)					54.25	254.70	0.831	
Parental Education (Some College) × Race/Ethnicity (Asian)					143.94	183.88	0.434	
Parental Education (Bachelor) × Race/Ethnicity (Asian)					241.60	165.06	0.143	
Parental Education (Post Graduate Degree) × Race/Ethnicity (Asian)					206.67	160.38	0.198	
Parental Education (HS Diploma/GED) × Race/Ethnicity (AIAN/NHPI)					491.49	205.56	0.017	*
Parental Education (Some College) × Race/Ethnicity (AIAN/NHPI)					278.92	197.45	0.158	
Parental Education (Bachelor) × Race/Ethnicity (AIAN/NHPI)					172.63	211.98	0.415	
Parental Education (Post Graduate Degree) × Race/Ethnicity (AIAN/NHPI)					778.50	311.03	0.012	*
Parental Education (HS Diploma/GED) × Race/Ethnicity (Other)					−102.06	73.80	0.167	
Parental Education (Some College) × Race/Ethnicity (Other)					−16.62	66.35	0.802	
Parental Education (Bachelor) × Race/Ethnicity (Other)					−126.67	79.73	0.112	
Parental Education (Post Graduate Degree) × Race/Ethnicity (Other)					−155.30	87.40	0.076	#
Parental Education (HS Diploma/GED) × Race/Ethnicity (Mixed)					−115.38	99.69	0.247	
Parental Education (Some College) × Race/Ethnicity (Mixed)					−84.07	86.30	0.330	
Parental Education (Bachelor) × Race/Ethnicity (Mixed)					−131.70	88.42	0.136	
Parental Education (Post Graduate Degree) × Race/Ethnicity (Mixed)					−159.03	87.66	0.070	#

# *p* < 0.1, * *p* < 0.05, ** *p* < 0.01, *** *p* < 0.001.

**Table 5:** Summary of mixed-effects regression models on the association between the right superior temporal cortical surface area and reading ability.

	b	SE	*p*	sig	b	SE	*p*	sig
	Model 1				Model 2			
**Outcome**: Reading Ability								
Right Superior Temporal Cortical Surface Area (mm2)	0.0016	0.0001	<0.001	***	0.00166	0.00017	<0.001	***
Parental Education (HS Diploma/GED)	2.08	0.35	<0.001	***	2.07	0.35	<0.001	***
Parental Education (Some College)	3.97	0.31	<0.001	***	3.97	0.31	<0.001	***
Parental Education (Bachelor)	5.57	0.33	<0.001	***	5.56	0.33	<0.001	***
Parental Education (Post Graduate Degree)	6.88	0.33	<0.001	***	6.88	0.33	<0.001	***
Age (Month)	0.21	0.01	<0.001	***	0.21	0.01	<0.001	***
Sex (Male)	−0.57	0.12	<0.001	***	−0.57	0.12	<0.001	***
Married Family	0.80	0.15	<0.001	***	0.80	0.15	<0.001	***
Race/Ethnicity (Black)	−2.21	0.21	<0.001	***	−0.78	1.45	0.590	
Race/Ethnicity (Asian)	1.56	0.38	<0.001	***	2.43	3.23	0.452	
Race/Ethnicity (AIAN/NHPI)	−0.93	0.67	0.163		−3.95	4.77	0.408	
Race/Ethnicity (Other)	−0.44	0.31	0.156		1.06	2.42	0.661	
Race/Ethnicity (Mixed)	−0.04	0.22	0.855		1.27	1.77	0.472	
Right Superior Temporal Cortical Surface Area (mm^2^) × Race/Ethnicity (Black)					−0.0004	0.0004	0.323	
Right Superior Temporal Cortical Surface Area (mm^2^) × Race/Ethnicity (Asian)					−0.0002	0.0008	0.789	
Right Superior Temporal Cortical Surface Area (mm^2^) × Race/Ethnicity (AIAN/NHPI)					0.0008	0.0012	0.524	
Right Superior Temporal Cortical Surface Area (mm^2^) × Race/Ethnicity (Other)					−0.0004	0.0006	0.532	
Right Superior Temporal Cortical Surface Area (mm^2^) × Race/Ethnicity (Mixed)					−0.0003	0.0005	0.456	

*** *p* < 0.001.

**Table 6:** Summary of mixed-effects regression models on the association between the left superior temporal cortical surface area and reading ability.

	b	SE	*p*	sig	b	SE	*p*	sig
	Model 1				Model 2			
**Outcome**: Reading Ability								
Left Superior Temporal Cortical Surface Area (mm2)	0.0016	0.0001	<0.001	***	0.0017	0.0002	<0.001	***
Parental Education (HS Diploma/GED)	2.08	0.35	<0.001	***	2.07	0.35	<0.001	***
Parental Education (Some College)	3.97	0.31	<0.001	***	3.97	0.31	<0.001	***
Parental Education (Bachelor)	5.57	0.33	<0.001	***	5.56	0.33	<0.001	***
Parental Education (Post Graduate Degree)	6.88	0.33	<0.001	***	6.88	0.33	<0.001	***
Age (Month)	0.21	0.01	<0.001	***	0.21	0.01	<0.001	***
Sex (Male)	−0.57	0.12	<0.001	***	−0.57	0.12	<0.001	***
Married Family	0.80	0.15	<0.001	***	0.80	0.15	<0.001	***
Race/Ethnicity (Black)	−2.21	0.21	<0.001	***	−0.78	1.45	0.590	
Race/Ethnicity (Asian)	1.56	0.38	<0.001	***	2.43	3.23	0.452	
Race/Ethnicity (AIAN/NHPI)	−0.93	0.67	0.163		−3.95	4.77	0.408	
Race/Ethnicity (Other)	−0.44	0.31	0.156		1.06	2.42	0.661	
Race/Ethnicity (Mixed)	−0.04	0.22	0.855		1.27	1.77	0.472	
Left Superior Temporal Cortical Surface Area (mm^2^) × Race/Ethnicity (Black)					−0.0004	0.0004	0.323	
Left Superior Temporal Cortical Surface Area (mm^2^) × Race/Ethnicity (Asian)					−0.0002	0.0008	0.789	
Left Superior Temporal Cortical Surface Area (mm^2^) × Race/Ethnicity (AIAN/NHPI)					0.0008	0.0012	0.524	
Left Superior Temporal Cortical Surface Area (mm^2^) × Race/Ethnicity (Other)					−0.0004	0.0006	0.532	
Left Superior Temporal Cortical Surface Area (mm^2^) × Race/Ethnicity (Mixed)					−0.0003	0.0005	0.456	

*** *p* < 0.001.

**Table 7:** Summary of mixed-effects regression models on the association between parental education and reading ability.

	b	SE	*p*	sig	b	SE	*p*	sig
	Model 1				Model 2			
**Outcome**: Reading Ability								
Parental Education (HS Diploma/GED)	2.10	0.34	<0.001	***	1.06	0.53	0.044	*
Parental Education (Some College)	4.03	0.31	<0.001	***	2.95	0.46	<0.001	***
Parental Education (Bachelor)	5.78	0.32	<0.001	***	4.77	0.46	<0.001	***
Parental Education (Post Graduate Degree)	7.18	0.32	<0.001	***	6.08	0.45	<0.001	***
Age (Month)	0.20	0.01	<0.001	***	0.20	0.01	<0.001	***
Sex (Male)	−0.15	0.12	0.194		−0.16	0.12	0.184	
Married Family	0.85	0.15	<0.001	***	0.82	0.15	<0.001	***
Race/Ethnicity (Black)	−2.39	0.21	<0.001	***	−4.47	0.68	<0.001	***
Race/Ethnicity (Asian)	1.44	0.38	<0.001	***	0.75	1.91	0.694	
Race/Ethnicity (AIAN/NHPI)	−0.95	0.66	0.153		−5.44	2.48	0.028	*
Race/Ethnicity (Other)	−0.56	0.31	0.069	#	−1.64	0.75	0.028	*
Race/Ethnicity (Mixed)	−0.14	0.22	0.510		−2.60	1.10	0.018	*
Parental Education (HS Diploma/GED) × Race/Ethnicity (Black)					1.88	0.80	0.020	*
Parental Education (Some College) × Race/Ethnicity (Black)					2.28	0.74	0.002	**
Parental Education (Bachelor) × Race/Ethnicity (Black)					2.42	0.82	0.003	**
Parental Education (Post Graduate Degree) × Race/Ethnicity (Black)					2.20	0.84	0.009	**
Parental Education (HS Diploma/GED) × Race/Ethnicity (Asian)					−1.91	3.37	0.571	
Parental Education (Some College) × Race/Ethnicity (Asian)					2.62	2.33	0.261	
Parental Education (Bachelor) × Race/Ethnicity (Asian)					0.47	2.03	0.816	
Parental Education (Post Graduate Degree) × Race/Ethnicity (Asian)					0.72	1.97	0.715	
Parental Education (HS Diploma/GED) × Race/Ethnicity (AIAN/NHPI)					3.07	2.80	0.273	
Parental Education (Some College) × Race/Ethnicity (AIAN/NHPI)					6.47	2.67	0.015	*
Parental Education (Bachelor) × Race/Ethnicity (AIAN/NHPI)					2.90	2.93	0.322	
Parental Education (Post Graduate Degree) × Race/Ethnicity (AIAN/NHPI)					5.19	3.68	0.159	
Parental Education (HS Diploma/GED) × Race/Ethnicity (Other)					2.58	0.99	0.009	**
Parental Education (Some College) × Race/Ethnicity (Other)					0.34	0.90	0.702	
Parental Education (Bachelor) × Race/Ethnicity (Other)					0.08	1.08	0.944	
Parental Education (Post Graduate Degree) × Race/Ethnicity (Other)					2.20	1.17	0.061	#
Parental Education (HS Diploma/GED) × Race/Ethnicity (Mixed)					1.77	1.33	0.182	
Parental Education (Some College) × Race/Ethnicity (Mixed)					2.48	1.15	0.032	*

# *p* < 0.1, * *p* < 0.05, ** *p* < 0.01, *** *p* < 0.001.

### 5.5. Figures

**Figure A4:**
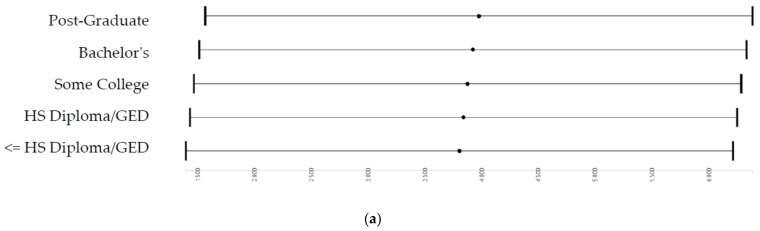

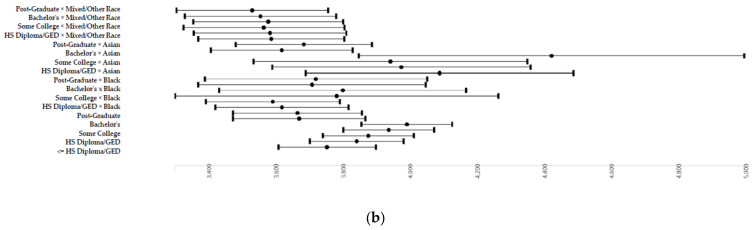
Association between parental education and children’s right superior temporal cortical surface area. In these figures, X represents children’s right superior temporal cortical surface area and Y represents parental education across racial groups. (**a**) Overall; (**b**) by race.

**Figure A5:**
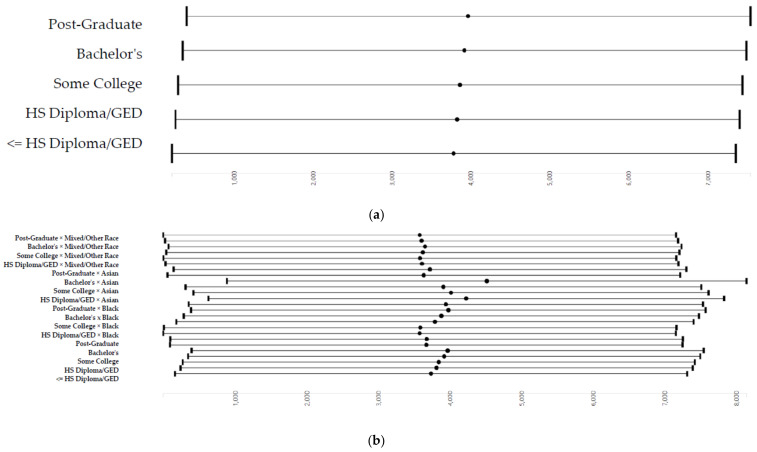
Association between parental education and children’s left superior temporal cortical surface area. In these figures, X represents children’s left superior temporal cortical surface area and Y represents parental education across racial groups. (**a**) Overall; (**b**) by race.

**Figure A6:**
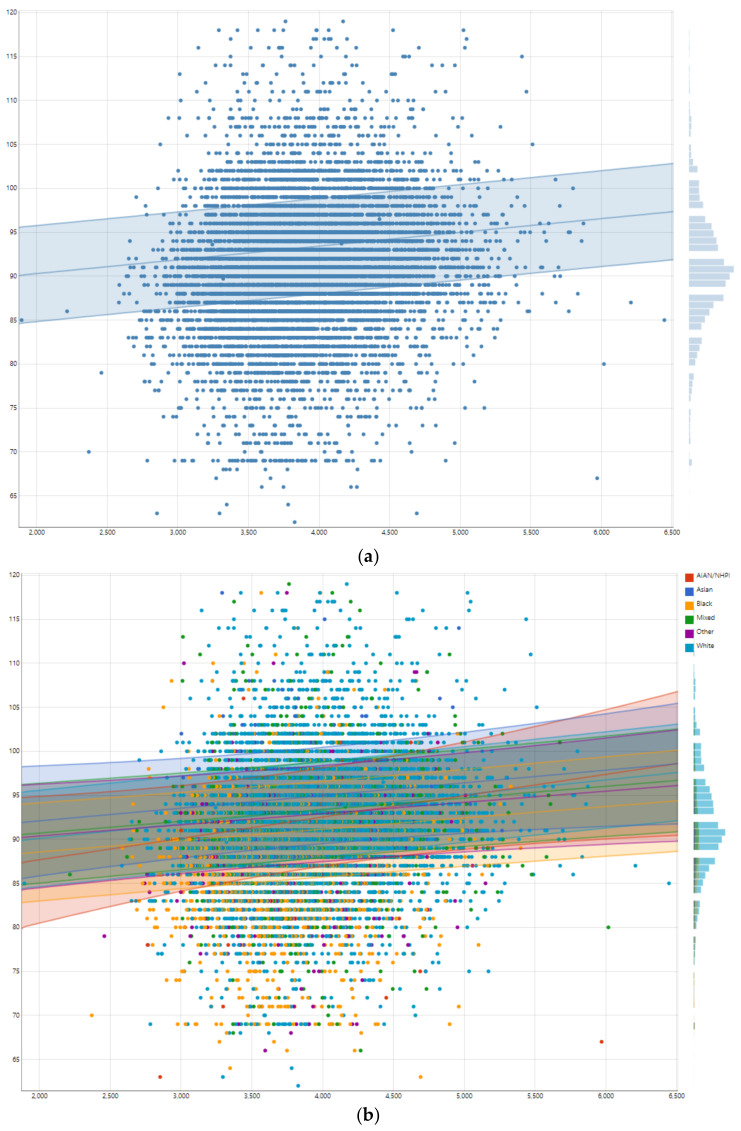
Association between the right superior temporal cortical surface area and children’s reading ability. In these figures, X represents children’s right superior temporal cortical surface area and Y represents children reading scores. (**a**) Overall; (**b**) by race.

**Figure A7:**
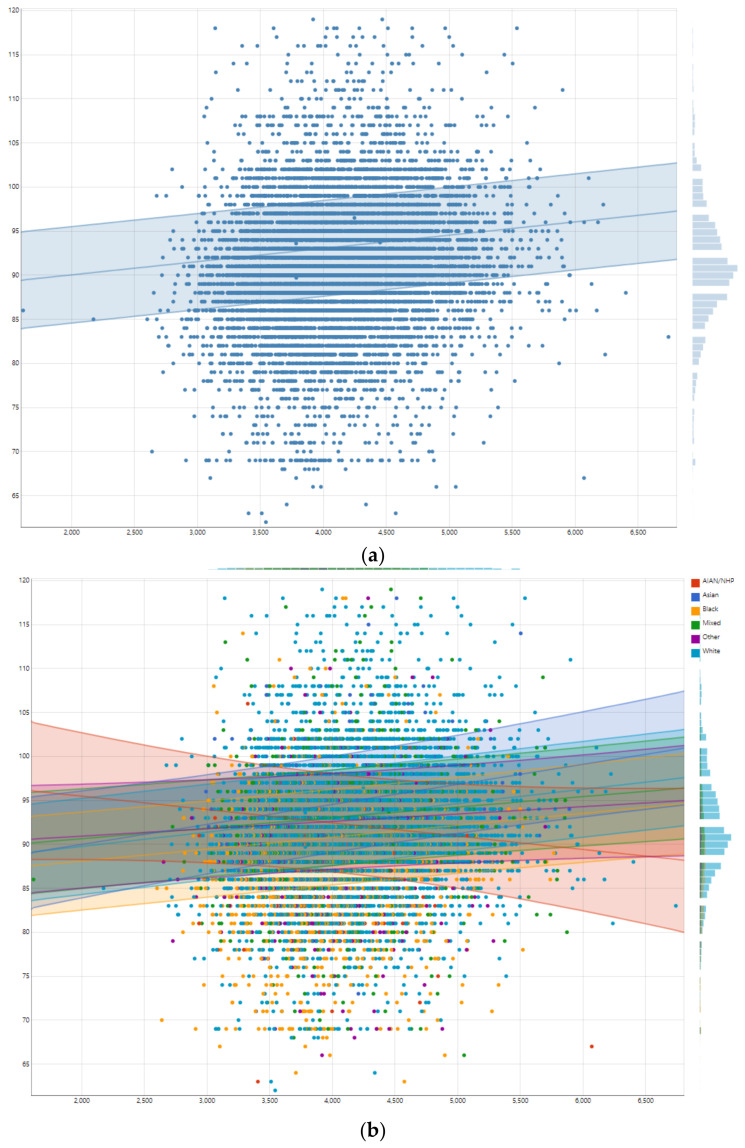
Association between the left superior temporal cortical surface area and parental education children’s reading ability. In these figures, X represents children’s left superior temporal cortical surface area and Y represents children reading scores. (**a**) Overall; (**b**) by race.

**Figure A8:**
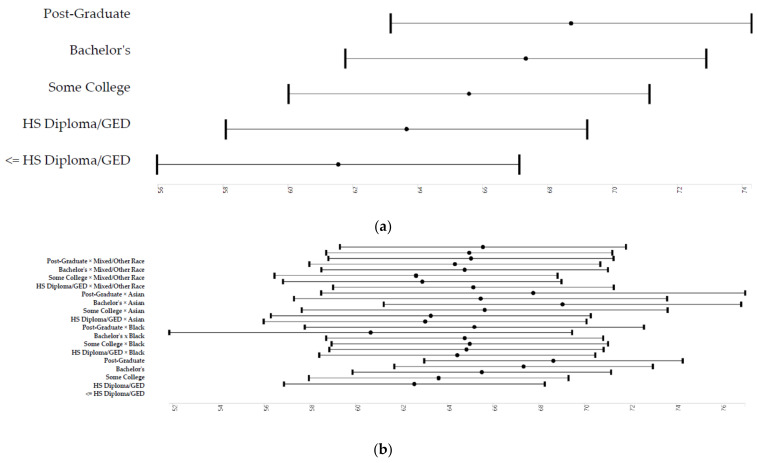
Association between parental education and children’s reading ability. In these figures, X represents children’s reading ability and Y represents parental education across racial groups. (**a**) Overall; (**b**) by race.

Appendix D includes a series of figures that depict the results shown in the above tables. Figure A4a, Figure A5a, Figure A6a, Figure A7a and Figure A8a showed that, in the pooled sample, parental educational attainment, children’s right and left superior temporal cortical surface area, and children’s reading ability were all positively correlated. As shown by Figure A4b and Figure A5b, race/ethnicity showed statistically significant interactions with parental educational attainment on children’s right and left superior temporal cortical surface area. These interactions suggest that high parental educational attainment has a smaller boosting effect on children’s right and left superior temporal cortical surface areas for black, other-race, and mixed-race children than white children (in line with MDRs). Figure A6b shows that the association between the right superior temporal cortical surface area and reading ability is different across various racial/ethnic groups. Figure A7b shows that the association between the left superior temporal cortical surface area and reading ability is weaker for AIAN/NHPI than white children (in line with MDRs). Figure A8b shows that the association between parental education and reading ability is weaker in white than black children (in contrast to MDRs).

### 5.6. Sensitivity Analysis (Robustness Check)

Given the replicability crisis of psychological and social studies [101,102,103,104] and the need to test the replicability of the results [105] overall and in the ABCD study [106], we ran several robustness checks to further test the validity of our results . These included: (1) models in the absence and presence of covariates, (2) models in the full sample and those that met imaging quality indicators, (3) models with and without a propensity score, and (4) models on the right and the left-brain hemispheres. Without exception, the results were replicated regardless of our modeling strategy. We present results for right and left-brain hemispheres, but not other outcomes. The results of our replications are available upon request.

## 6. Discussion

Our results support our hypotheses about MDRs, as evidenced by our finding that parental educational attainment, the superior temporal cortical surface area, and reading ability are all correlated in 9–10-year-old children, but the magnitude of the associations differed across racial/ethnic groups. While the associations between parental educational attainment and children’s right and left superior temporal cortical surface areas, as well as the association between children’s left superior temporal cortical surface areas and children’s reading ability, are weaker in some non-white racial/ethnic groups of children than their white peers, the association between parental education and reading ability is unexpectedly stronger in black than white children.

The weaker observed associations in non-white than white children are consistent with other analyses of MDRs, using ABCD study data for a wide range of neurocognitive domains [102], supported by brain imaging [41]. To the best of our knowledge, the MDRs reported here were not known before: (1) the link between parental educational attainment and right and left superior temporal cortical surface areas and (2) the link between children’s left superior temporal cortical surface areas and children’s reading ability. These MDRs were found for various non-white groups of children, such as black, other-race, mixed-race, or AIAN/NHPI. This observation is consistent with the notion that MDRs are not specific to black children because we found them for all marginalized groups [56,57,67,106,107].

The observation of a stronger association between parental education and reading ability in black than white children did not support our hypotheses. However, this finding may be due to compensatory effects. For example, black parents may understand, from generations of disadvantage, that they must work twice as hard as white people to achieve half as much [70]. Nevertheless, structural factors are stacked against them, in terms of fewer educational resources, if they live in a district with predominantly non-white families [10], teacher discrimination [103], differential disciplinary actions [104], differential teacher expectations and grading [104], differential teacher preparedness [105], and less access to academic enrichment opportunities [106]. More educated black families may be more likely than their white counterparts to help their children in ways that are in their control, such as reading to them and supporting activities that may help reading more than other areas, such as math and science, which may require more time and resources. In other words, reading is easier to do as a family than math- or science-related enrichment and is therefore less influenced by MDRs. This interpretation is somewhat supported by the fact that we did find differences in child brain structures. This reading ability result may also indicate that families can overcome the negative effects of MDRs when they engage in enrichment activities of their own, but this does not change the fact that fundamental structural change is necessary because such activities are essentially a tax for black families as a way to keep up with their more privileged white counterparts. In some recent ABCD study data analyses, the SES effects on neurocognitive domains are weaker for black than white children [60,77,105]. In one study, the effects of parental education on mental rotation (a specific cognitive task) was weaker in black than white children [107]. In another analysis, the effect of parental education on inhibitory control was weaker for black than white children [102]. In another analysis of ABCD study data, age had weaker effects of inhibitory control for black than white children [63]. Similar results were found for the effects of age on children’s dimensional card changing activities [62].

In addition to the ABCD study data [102], similar MDRs are shown in several national studies, such as the Fragile Families and Child Wellbeing Study (FFCWS) [69,108,109,110,111], Population Assessment of Tobacco and Health (PATH) [47,112,113,114], Education Longitudinal Study (ELS) [115], Monitoring the Future (MTF) [53], and National Survey of American Life (NSAL) [50], all showing weaker effects of SES indicators on outcomes for black children compared to white children. For example, in the PATH data, SES effects on future tobacco use [47], in the FFCWS study, SES effects on ADHD, impulsivity, obesity, and self-rated health [69,108,109,110,111], in ELS, SES effects on school quality [115], and in MTF, SES effects on school performance were all weaker for black than white children [53]. In a longitudinal study of Flint, for MI adolescents, the effect of having married parents on subsequent anxiety symptoms was weaker for black than white children who were transitioning to young adulthood [116]. In the NSAL-adolescents data, high SES positively correlated with depression for black boys [50]. Thus, the MDRs reported here (for two of three aims) are consistent with past research and not specific to a particular brain mechanism. Our findings add to the growing body of empirical work that suggests that these patterns are nearly universal. They also support that MDR-related disparities result from structural and contextual factors and are not due to deficient culture or behaviors.

In our study, the MDR hypothesis was found for black, other, mixed-race, and AIAN/NHPI families. Researchers have documented MDRs for black [48], Hispanic [48], Asian [56], AIAN/NHPI [117], and even marginalized white families [57]. These MDRs are also relevant to other types of marginalization based on sexual orientation and nativity (immigration) status. Individuals with LGBT [67,118] and immigrant status [64,65,119,120] show weaker health effects of SES than those with a non-LGBT and non-immigrant status. This non-specificity of MDRs across measures of the SES resource, outcomes, populations, and age groups suggests that MDRs are likely due to structural racism and social stratification, which cause disparities across domains [57]. Again, this universality of the patterns suggests that any deviation from majority norms and expectations (e.g., whiteness, straightness) comes with a penalty for the marginalized group. The privileges associated with the majority expectations include policy and laws, resource distribution, and opportunities for educational and economic advancement that contribute to inequality, and the odds are stacked against non-majority individuals, even at similar SES levels. Our results suggest that MDRs are strongest for black people whose American experience has been centuries and generations in the making [121]. These MDRs can reflect the existing structural racism in the US [83,84]. Another explanation of this phenomenon is that, in the absence of government interventions that undo some of the inequalities, the rich become richer and the poor, marginalized, and non-white become poorer [74]. This is because the Haves lead the making and take advantage of policies rather than the Have-Nots [74]. Unless some equalizing forces are in place, we cannot expect inequalities to narrow over time [122]. A recent report shows that governmental spending minimizes [123,124,125] the existing inequalities. For example, some research suggests that the Affordable Care Act (ACA) has reduced inequalities [126,127,128,129,130,131,132], in part because some of these applied policies may have larger effects on the underserved than privileged populations [133].

Reduced effects of parental education in black and white families might be a function of labor market discrimination and other unequal processes that hinder black people across SES and class lines [134]. Upward social mobility is very taxing and costly for black families, particularly black men [70]. Highly educated black people work in worse jobs than their white counterparts with similar education levels and receive lower salaries [135,136]. As a result, highly educated black people generate less wealth than white people with similar education [45]. Zajacova has argued that, instead of being an equalizer, education contributes and even generates inequality across populations [137,138]. These MDRs generate inequality without racist actors. This is why we interpret MDRs as indicators of structural racism. In the presence of MDRs, inequalities are regenerated, and poor outcomes are transitioned from one to the next generation. As such, MDRs can explain why inequalities persist over time, despite the overall improvement in SES and health [139].

In high SES families, stress remains at a higher level in black than white families [135,140]. Highly educated black families are more likely to remain in neighborhoods with high stress [69] and poor schools [115] than high SES white people. When high SES black families move for opportunity, they are heavily discriminated against at schools [141] and workplaces [142]. Such discrimination reduces black children’s GPA [103], mental health [143,144,145], and physical health [146,147,148]. Surprisingly, SES and discrimination are positively associated in black families [149,150,151]. The same association is reversed for white families [152]. As a result of all these structural inequalities, non-white children show worse than expected outcomes in high SES families, a pattern opposite to high SES white families. As explained, these processes are social, not biological, suggesting that these MDRs are shaped by racism, not genetics [43,44,45].

Our results also suggest that race needs to be conceptualized as a proxy of racism, social stratification, adversity, differential treatment, the legacy of slavery, Jim Crow, and other processes, such as interpersonal discrimination. In our framework, race is a proxy of social forces and constraint choices, rather than genes. We argue that racism alters the SES effects, so we observe race by SES interactions, as discussed here. As such, inequalities are modifiable via effective public policy that contributes to a more fair and equal American society. That said, MDRs indicate a social and physical context, educational opportunities, access to resources, and daily lived experiences of non-whites across SES levels and class lines. SES differently changes experiences and opportunities for whites and non-whites and other non-majority groups. As one’s race and ethnicity are more apparent than one’s SES, high SES non-whites are frequently discriminated against in society, a pattern which is also seen in schools [103]. Thus, we need to address societal barriers that diminish parental education’s marginal returns for middle-class racial and ethnic minority families if we want to minimize the racial/ethnic gap in children’s brain development, school achievement, and reading ability. Social and public policies need to go beyond equal access by addressing structural and societal barriers that hinder minority families and their children. Multi-level barriers, such as social stratification, unfair policing, poor schooling, segregation, racism, and discrimination, are common in the daily lives of communities of color. These experiences take a tremendous toll on their children’s brain and academic development, even if their SES is average or higher. Consequently, solutions to MDRs must go beyond individual victim-blaming approaches and require multi-level policy solutions that begin to break down the structural factors that underlie the inequality in the first place.

As this paper shows, we have distanced ourselves from any biological argument, such that brain structure is under the influence of race as a biological variable. However, there are modifiable biological changes that may occur under the influence of racism, toxic stress, oppression, and adversity. Hundreds of years under slavery, poverty, intimidation, discrimination, and murder have likely had an enormous effect on the hypothalamic-pituitary-adrenal (HPA) axis, inflammation, allostatic load, epigenetics, and other mechanisms that mediate the effect of racism on the body [153]. For example, a growing body of research has documented epigenetic changes as a function of discrimination, stress, and racism [154,155,156]. Environments, such as lead, particular matters, and other toxins, also have epigenetic effects [154,155,156]. Pollutions in air and water, poor nutrition, and toxic stress can increase the epigenetic risk of black children and adults [154,155,156]. Arline Geronimus has worked extensively on biological changes that occur in response to adversity [157].

Still, it is unclear how these racism-related biological changes would explain our findings of racial variation in the associations between parental education, brain, and reading ability among American children. We still believe that a straightforward theory is a social mechanism: As a result of racism, living conditions of highly educated black and white families are qualitatively different, so parental education has a differential association with child brain and child reading, because black children are exposed to less enriching environments than their white counterparts, given social stratification and segregation [158]. Addressing racism may also help black parents engage in more enrichment activities, so we will see a higher brain development and a better reading ability of black children in highly educated families. Currently, highly educated black families are paying the extra “Black tax” [159], which may hinder their children’s opportunities to use enriched activities that can help to overcome the reading deficiencies seen amongst so many black children.

This paper acknowledges the MDR framework shown in so many studies investigating educational outcomes among children of color in the US [43,44]. As such, this paper provides additional evidence showing that significant effects of structural racism are still evident when SES is controlled. Structural racism may shape how parental education, brain structure, and reading ability correlate in American children. 

At the same time, we do not deny the enormous progress made over the past centuries. We identify a great degree of progress being made among black families with higher education. The US has had a black president, there are more black people in congress and the senate than ever, Black Lives Matter receives enormous support, people of all colors and races participate in demonstrations for black people’s rights, and police officers were recently convicted for the killing of a Black man [160]. Thus, the US has made considerable progress and we do not deny it [161]. We simply say that this progress is not enough, and there is a need for additional work to be done [162].

Our results support past research that MDRs are relevant for black individuals and the idea that deviation from whiteness might be associated with a reduced gain in outcomes expected to follow access to SES resources [163]. Structural racism may affect biology, as well allostatic load and the HPA axis [153]. More research is needed on how racism gets under the skin and the role of toxins, allostatic load, and the HPA axis in this regard.

## 7. Limitations

Several limitations of our study should be noted. First, our study design is cross-sectional. As such, we cannot conclude or infer any causal effects between race, SES, brain development, or reading ability. Second, the sample was imbalanced across race/ethnic groups. Most participants were white, and a small proportion was Asian, AIAN/NHPI, and other-race. Third, the sample was not nationally representative. As such, we cannot generalize the results to all US children. Fourth, we only used a single sMRI indicator rather than multiple sMRI, dMRI, or fMRI measures across ROIs and brain structures. Fifth, we did not use other sources of data, such as school records of reading scores. Nevertheless, our study adds to the growing body of work on MDRs by focusing attention to brain structures in a large sample from across the US, application of a widely accepted and validated neurocognitive measure, highly homogenous samples in terms of age and development (resulting in high internal validity), and multiple robustness checks, suggesting that our results were not anomalous in the data. Our use of propensity score also increases the generalizability of our results and comparability of race/ethnic groups.

## 8. Future Directions

There is plenty of research to support the adverse effects of racism on children’s development, but there are many other factors that may contribute to poor reading ability in children that should be considered. In addition to racism, marginalization is also linked to poverty and adverse childhood experiences, as well as a wide range of other exposures, such as toxins and all types of stress. In this study, we focused on the indirect effect of race when SES is controlled. Future research may focus on other factors, such as poverty, toxins, epigenetics, HPA axis, allostatic load, adverse childhood experiences, stress, housing and food insecurity, education quality, neighborhood conditions, and other explanatory factors.

## 9. Conclusions

In summary, associations between parental education and children’s superior temporal cortical surface area were weaker for black, other-race, and mixed-race children than white children. The effect of the left superior temporal cortical surface area on reading ability was also weaker in AIAN/NHPI children than white children. Most of these findings are in line with MDRs. Using a MDR lens to understand the finding that parental educational attainment and children’s reading ability showed the opposite pattern (i.e., stronger, not weaker, effects for black compared to white children) suggests that reading may be one skill for children that black families have some control over (versus structural inequities) and therefore can take time to help their children with their reading ability. However, this tax creates other inequities in what black families may have to do to maintain some semblance of equitable developmental opportunities. Our study suggests that research into the socioecological and environmental mechanisms that explain why MDRs emerge for some predictors, processes, and outcomes, but not others, is necessary.

## Figures and Tables

**Table 1:** Descriptive data for the overall sample and by race/ethnicity.

Level	All		White		Black		Asian		AIAN/NHPI		Other-Race		Mixed-Race		*p*	
		Weighted		Weighted		Weighted		Weighted		Weighted		Weighted		Weighted		Weighted
*n*	10,817		7091		1646		246		68		468		1298			
Age (Month)	119.04 (7.47)	119.32 (7.49)	119.10 (7.49)	119.38 (7.50)	118.86 (7.27)	119.14 (7.28)	119.54 (7.79)	119.91 (7.77)	118.00 (7.30)	118.60 (7.20)	118.75 (7.60)	118.96 (7.61)	118.94 (7.48)	119.13 (7.59)	0.437	0.478
Right Superior Temporal Surface Area (mm^2^)	3911.63 (456.34)	3897.79 (457.60)	3951.42 (454.51)	3932.68 (457.36)	3755.05 (430.82)	3742.63 (428.40)	3907.72 (429.86)	3901.32 (429.11)	3931.47 (523.94)	3934.30 (584.94)	3824.22 (430.71)	3815.88 (436.33)	3924.04 (461.85)	3919.07 (452.95)	<0.001	<0.001
Left Superior Temporal Surface Area (mm^2^)	4124.01 (507.98)	4110.51 (510.07)	4168.12 (507.51)	4149.13 (510.35)	3967.99 (477.53)	3957.39 (477.14)	4093.99 (539.90)	4082.89 (536.78)	4092.22 (559.98)	4070.71 (610.38)	4026.10 (470.90)	4018.28 (471.30)	4123.51 (508.28)	4129.08 (501.39)	<0.001	<0.001
Reading Ability	90.96 (6.85)	90.68(6.94)	91.76 (6.45)	91.44 (6.57)	87.35 (7.02)	87.00 (7.02)	94.67 (6.43)	94.52 (6.50)	88.41 (7.64)	87.81 (8.22)	88.79 (6.89)	88.95 (7.05)	91.39 (6.96)	90.50 (6.95)	<0.001	<0.001
Parental Education																
<High School Diploma	470 (4.3)	(5.4)	194 (2.7)	(3.7)	137 (8.3)	(9.6)	6 (2.4)	(2.1)	6 (8.8)	(9.7)	92 (19.7)	(19.5)	35 (2.7)	(4.4)	<0.001	<0.001
High School Diploma/Graduate Equivalency Degree	978 (9.0)	(10.7)	362 (5.1)	(6.7)	404 (24.5)	(27.1)	4 (1.6)	(2.1)	16 (23.5)	(26.7)	107 (22.9)	(23.5)	85 (6.5)	(9.2)		
Some College	2799 (25.9)	(29.7)	1514 (21.4)	(26.8)	647 (39.3)	(40.6)	18 (7.3)	(7.9)	29 (42.6)	(40.8)	162 (34.6)	(32.8)	429 (33.1)	(42.5)		
Bachelor	2799 (25.9)	(24.4)	2091 (29.5)	(27.9)	230 (14.0)	(12.5)	65 (26.4)	(27.0)	14 (20.6)	(19.7)	63 (13.5)	(13.8)	336 (25.9)	(21.9)		
Post Graduate Degree	3771 (34.9)	(29.8)	2930 (41.3)	(34.9)	228 (13.9)	(10.3)	153 (62.2)	(61.0)	3 (4.4)	(3.1)	44 (9.4)	(10.4)	413 (31.8)	(21.9)		
Sex																
Female	5175 (47.8)	(48.9)	3337 (47.1)	(48.2)	824 (50.1)	(51.2)	127 (51.6)	(51.9)	37 (54.4)	(58.4)	213 (45.5)	(45.7)	637 (49.1)	(50.7)	0.095	0.095
Male	5642 (52.2)	(51.1)	3754 (52.9)	(51.8)	822 (49.9)	(48.8)	119 (48.4)	(48.1)	31 (45.6)	(41.6)	255 (54.5)	(54.3)	661 (50.9)	(49.3)		
Married Family																
No	3402 (31.5)	(38.1)	1506 (21.2)	(29.5)	1156 (70.2)	(76.8)	35 (14.2)	(15.6)	33 (48.5)	(45.5)	224 (47.9)	(51.1)	448 (34.5)	(45.4)	<0.001	<0.001
Yes	7415 (68.5)	(61.9)	5585 (78.8)	(70.5)	490 (29.8)	(23.2)	211 (85.8)	(84.4)	35 (51.5)	(54.5)	244 (52.1)	(48.9)	850 (65.5)	(54.6)		

**Table 2:** Variance in outcomes explained by each model.

	Independent Variable: Parental Education			
	**Outcome**: Superior Temporal Cortical Surface Area			
	Right		Left	
	Model 1 Main Effects	Model 2 Main Effects + Interactions	Model 1 Main Effects	Model 2 Main Effects + Interactions
	Effect Size	Effect Size	Effect Size	Effect Size
*n*	10817	10817	10817	10817
R-squared	0.11395	0.12775	0.16911	0.17197
ΔR-squared	0.00218 (0.22%)	0.03066 (3.07%)	0.00975 (0.98%)	0.02859 (2.86%)
	**Independent Variable**: Superior Temporal Cortical Surface Area			
	**Outcome**: Reading Ability			
	Right		Left	
	Model 1 Main Effects	Model 2 Main Effects + Interactions	Model 1 Main Effects	Model 2 Main Effects + Interactions
*n*	11,117	11,117	11,117	11,117
R-squared	0.17809	0.17825	0.17914	0.18
ΔR-squared	0.00151 (0.15%)	0.01571 (1.57%)	0.00278 (0.28%)	0.01781 (1.78%)
	**Independent Variable**: Parental Education			
	**Outcome**: Reading Ability			
	Model 1 Main Effects	Model 2 Main Effects + Interactions		
*n*	11,440	11,440		
R-squared	0.17007	0.17289		
ΔR-squared	0.06144 (6.14%)	0.092 (9.2%)		

**Figure A1:**
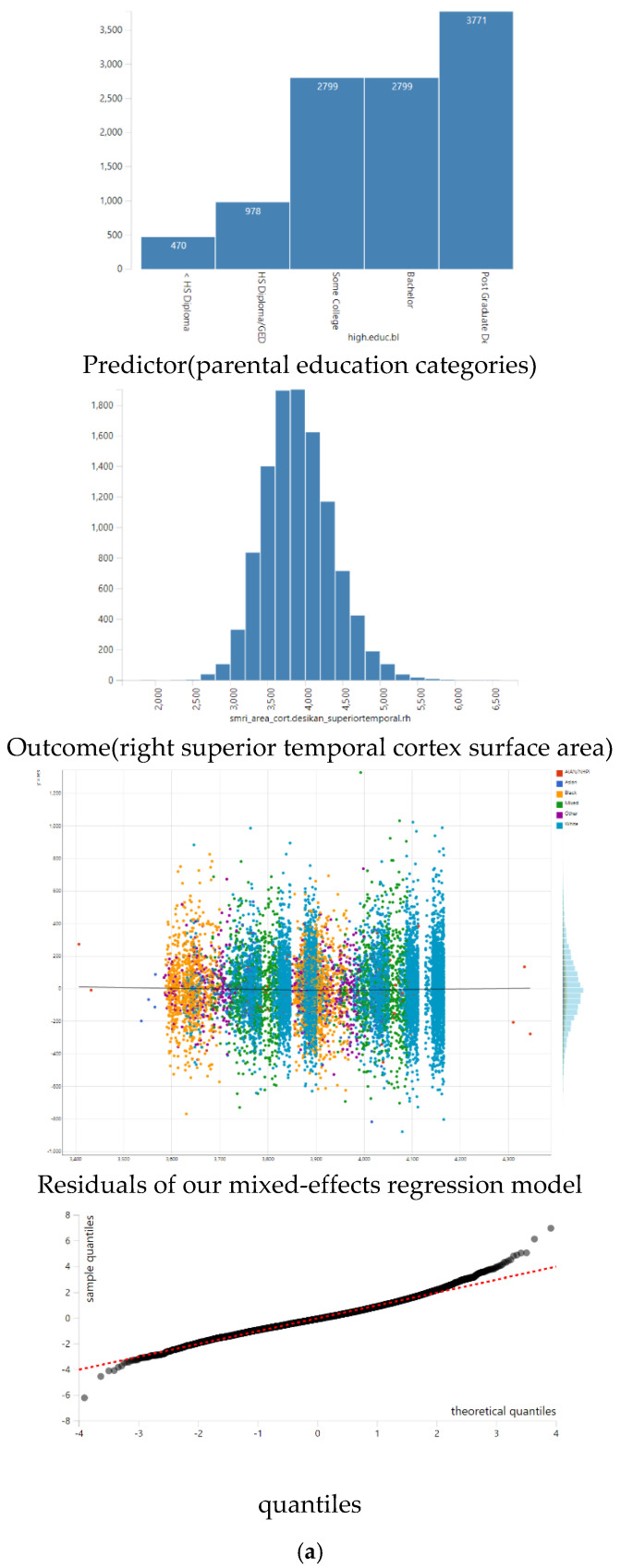

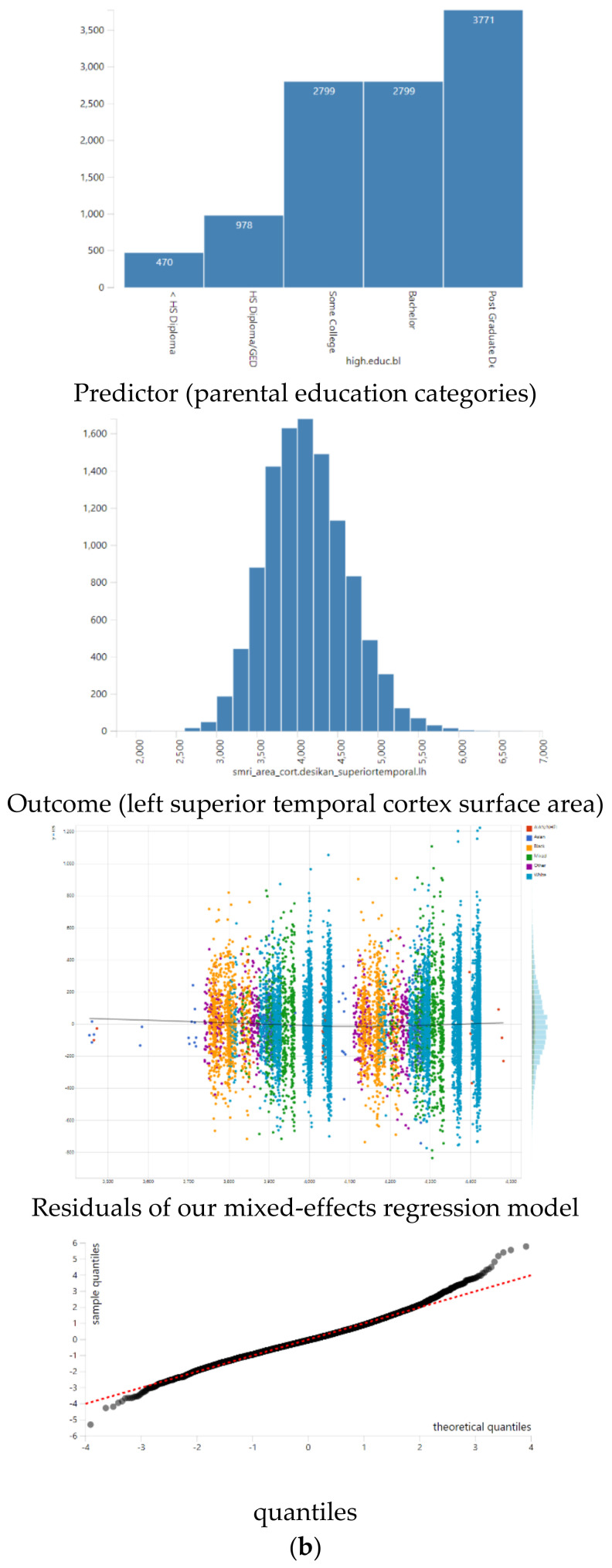
(**a**) Right superior temporal cortical surface area; (**b**) Left superior temporal cortical surface area.

**Figure A2:**
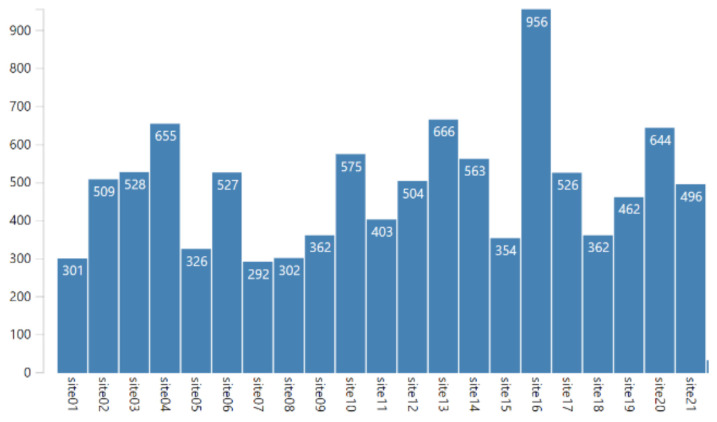
Distributions of the Study Variables and Testing the Model Assumptions.

**Figure A3:**
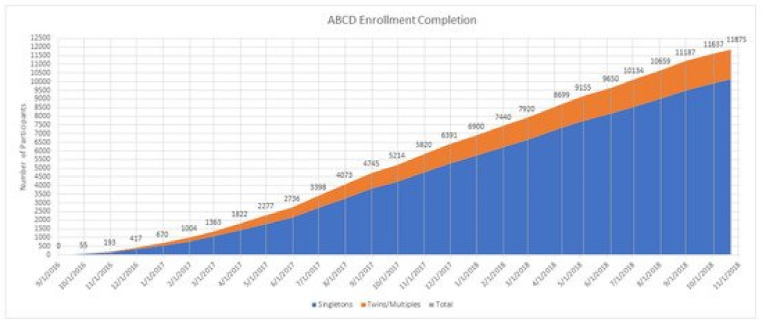
ABCD Enrollment Completion.

## Data Availability

Data are available at https://nda.nih.gov/abcd (accessed on 5 May 2021).
